# The Effect of Bariatric Surgery on Hypertension Outcomes: A Retrospective Cohort Study

**DOI:** 10.1038/s41371-025-01063-z

**Published:** 2025-08-20

**Authors:** Jesse E. Passman, Amanda Bader, Nadim Mahmud, Kristoffel R. Dumon, Heather Wachtel, Feibi Zheng, Jordana B. Cohen

**Affiliations:** 1 Department of Surgery, Perelman School of Medicine, University of Pennsylvania, Philadelphia, PA USA; 2 Leonard Davis Institute, University of Pennsylvania, Philadelphia, PA USA; 3 Division of Gastroenterology, Perlman School of Medicine, University of Pennsylvania, Philadelphia, PA USA; 4 Global Health Economics & Outcomes Research, Intuitive, Sunnyvale, CA USA; 5 Michael E. DeBakey Department of Surgery, Baylor College of Medicine, Houston, TX USA; 6 Renal-Electrolyte and Hypertension Division, Department of Medicine, Perelman School of Medicine, University of Pennsylvania, Philadelphia, PA USA; 7 Department of Biostatistics, Epidemiology, and Informatics, Perelman School of Medicine, University of Pennsylvania, Philadelphia, PA USA

**Keywords:** Hypertension, Risk factors, Disease prevention

## Abstract

Metabolic and bariatric surgery (MBS) is an effective treatment for obesity and metabolic syndrome. Evidence regarding the impact of MBS on hypertension outcomes is limited by short-term follow-up. Thus, this retrospective cohort study was designed to compare blood pressure (BP) control, number of antihypertensive medications (AHMs), development of apparent treatment resistant hypertension (ATRH), and remission of hypertension between patients treated with and without MBS. Adults with BMI ≥ 35 kg/m^2^ and a new diagnosis of hypertension receiving care within the Veterans Health Administration system from 2000–2019 were included. Generalized estimating equations and time-updated Cox models with inverse probability of treatment weighting to address time-updated confounding were used. Over a median follow-up of 5.1 years, 183702 patients with BMI ≥ 35 kg/m^2^ and hypertension were managed medically and 3965 were managed surgically. At baseline, those who underwent MBS were more likely to be women than men (22 vs. 10%). Patients treated surgically demonstrated significantly better BP control over time, with an average 5.4 mm Hg (95% CI 4.9–5.9) lower systolic BP and 1.8 mm Hg (95% CI 1.5–2.1) lower diastolic BP. Compared to patients treated medically, those who received MBS had 32% higher likelihood of complete AHM discontinuation (95% CI 1.23–1.42). Patients treated with MBS were 14% less likely to develop ATRH (95% CI 0.78–0.95). Overall, among patients with obesity and hypertension, treatment with MBS was associated with durably improved BP control compared to medical management, including lower systolic and diastolic BPs, higher AHM cessation, and lower rates of ATRH.

## Background

Hypertension is one of the most common and morbid medical conditions in the United States. Hypertension affects nearly 50% of the adult population and contributes to nearly 300 000 deaths per year through its role in adverse cardiovascular outcomes [1, 2]. While the incidence of hypertension-related morbidity initially declined in the 2000s with meaningful improvements in smoking cessation, aggressive risk factor modification, and widespread utilization of cardio-preventive medications, recent years have seen a deceleration of these declines. Many postulate this lost progress is directly attributable to increases in the rates of obesity and its associated metabolic syndrome [2, 3]. Indeed, obesity rates in the United States have continued to rise, with predictions that almost fifty percent of the adult population will have obesity by 2030 [4, 5]. Given the clear causal relationship between obesity and elevated blood pressure [1, 6], it follows that as obesity rates have increased, so too have rates of hypertension and other obesity-attributable cardiovascular, metabolic, and renal diseases [6]. Obesity not only influences hypertension incidence, it is also negatively associated with blood pressure control in patients with this diagnosis [7]. Rates of well-controlled blood pressure amongst all patients with hypertension are about 40%; in patients with obesity, they are only 18% [1, 7].

Metabolic and bariatric surgery (MBS) offers one potential solution to the increase in hypertension associated with obesity. Not only does MBS produce durable weight loss, but it also leads to improved control of a variety of downstream metabolic and cardiovascular diseases, including hypertension [8]. Nearly two-thirds of patients who undergo MBS experience improvement in their blood pressure control, with some analyses suggesting up to half of these patients will have complete resolution [9–11]. MBS patients naïve to antihypertensive medications (AHMs) are less likely to start taking them, whereas those on AHMs already are more likely to discontinue them [11, 12]. Further, rates of apparent treatment resistant hypertension (ATRH) are significantly lower in patients who undergo MBS [12]. However, most of these data are from retrospective studies with highly selected patient populations, limited follow-up duration, and non-specific endpoint definitions. One significant exception is the GATEWAY (Gastric Bypass to Treat Obese Patients With Steady Hypertension) Trial, which prospectively randomized 100 patients to Roux-en-Y gastric bypass (RYGB) plus medical therapy or medical therapy alone with AHM use as the primary endpoint [13]. With five year longitudinal follow up, this trial found that RYGB patients were significantly more likely to reduce AHM use by >30% compared to patients treated with medical therapy alone [13–15]. Nonetheless, longer term evaluation of hypertension outcomes across large real world populations of patients treated with MBS is necessary to understand the durability and external validity of these findings.

In this retrospective cohort study, we assessed rates of adequate blood pressure control, AHM use, and ATRH based on receipt of MBS over a period of 17 years amongst Veterans with obesity and hypertension. Specifically, we aimed to characterize the relative effect of MBS on blood pressure, AHM discontinuation, and likelihood of developing ATRH compared to patients who did not undergo MBS. We hypothesized that patients who received MBS would experience improved blood pressure control, lower AHM prescription rates, and lower likelihood of developing ATRH.

## Materials and methods

### Data Source

This study was conducted using a national cohort of approximately 9 million Veterans receiving medical care from the Veterans Health Administration (VHA), the largest integrated health system in the United States. The VHA Corporate Data Warehouse contains national electronic health record data including detailed demographic, clinical, laboratory, and pharmacy fill records. This study was approved by the Institutional Review Board (IRB) of the Corporal Michael J. Crescenz Veterans Affairs Medical Center in Philadelphia, PA. As this was a retrospective study with no intervention and no way to reidentify participants in the cohort, a waiver of informed consent was obtained from the IRB. Strengthening the Reporting of Observational Studies in Epidemiology (STROBE) guidelines were followed through the design and conduct of the study.

### Cohort Derivation

The study utilized the VHA Antihypertensives in Obesity Management Cohort, a dataset containing detailed, time-updated demographic, clinical, and outcomes data from over one million Veterans newly initiated on antihypertensive medication from 2000-2019 and followed through 2020 [16]. Hypertension diagnosis was determined based on the presence of both an International Classification of Diseases (ICD) (9^th^ or 10^th^ edition) billing code for hypertension (Table S1) and the presence of a minimum of two consecutive prescriptions for AHM (Table S2), with no prior evidence of antihypertensive medication use. Only individuals with at least one primary care visit in the year prior to initiating antihypertensive therapy were included. The index date for all patients is defined as date of their first antihypertensive medication fill.

**Fig. 1:**
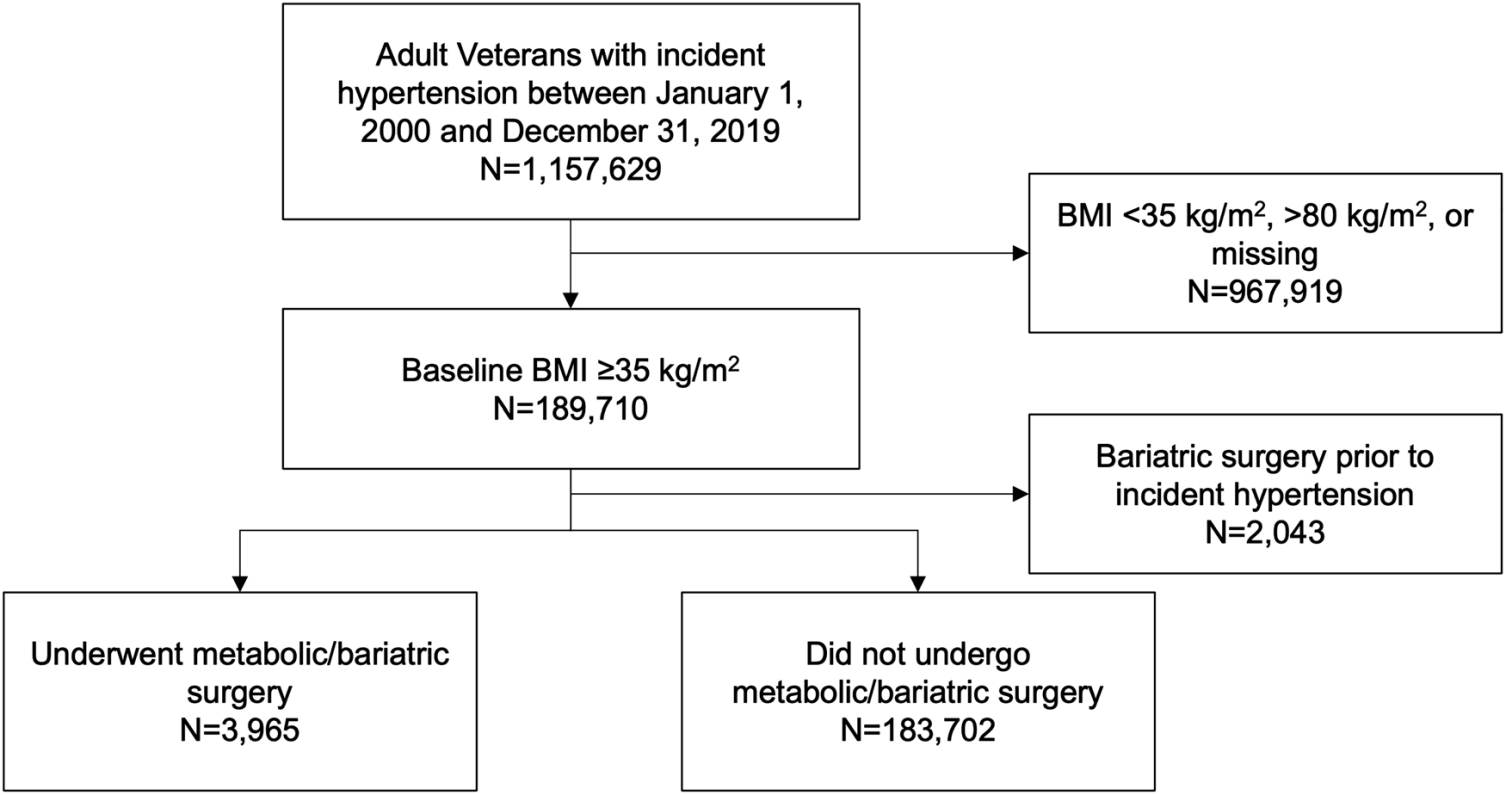
Cohort derivation for included patients.

For the current study, individuals were included who were ≥18 years of age with a BMI ≥ 35 kg/m^2^ at the time of developing new hypertension, as shown in Fig. 1. Individuals were excluded who were missing BMI, had BMI outside of clinically likely range ( ≥80 kg/m^2^), and who underwent MBS prior to meeting inclusion criteria.

### Primary Exposure

The primary exposure was time-updated MBS status. Included patients were divided into two groups for comparison of baseline characteristics: patients who received MBS on or after index date (the “surgical cohort”) and patients who did not receive MBS on or after the index date (the “medical cohort”). The surgical cohort included individuals who underwent MBS (RYGB, sleeve gastrectomy, duodenal switch, gastric band, or unknown type), which was defined by the presence of a Current Procedural Terminology (CPT-4) code, ICD-9 or ICD-10 code, or a VHA-specific procedure code (i.e., “stop code”) indicating an encounter for MBS (Table S1). The medical cohort included patients without evidence of MBS before or after index date.

### Covariates

We determined baseline and time-updated covariates using well-established algorithms [16–20]. Baseline comorbidities were defined by either one inpatient or two outpatient diagnostic codes on or before the index date (Table S1). Time-updated comorbidities were carried forward from baseline and were updated if any new diagnostic information was documented in 6-month intervals following the index date. VHA pharmacy fill data was used to determine baseline AHM use and time-updated AHM use in 6-month intervals following the index date. Individuals with pharmacy fills covering at least half of a given interval were considered to be taking the medication. All blood pressure measurements were restricted to those that were measured in outpatient primary care or selected internal medicine subspecialty practices that typically manage hypertension in order to maximize the reliability of blood pressure measurements and the likelihood of repeat measurements if an initial measurement was suspected to be unreliable [21]. Baseline systolic and diastolic blood pressures were defined as the mean of all eligible blood pressure values during the year prior to the index date. Baseline BMI and estimated glomerular filtration rate (eGFR; calculated using the 2021 CKD-EPI equation [22]) values were defined as the values closest to the index date, limited to those collected in the year prior to the index date. Time-updated blood pressures and BMIs were defined as the mean of all eligible values during each six-month interval following the index date.

### Outcomes

Included patients were followed until their final documented follow-up encounter through December 31, 2020, or death. The primary outcome was blood pressure control, defined by average outpatient systolic and diastolic blood pressure over the duration of follow-up. Secondary outcomes included average number of distinct AHM classes prescribed over time, likelihood of complete AHM discontinuation, and incidence of ATRH, defined as uncontrolled blood pressure while taking three AHMs or use of at least four AHMs regardless of blood pressure control [16, 23].

### Statistical Analyses

Baseline demographic and clinical characteristics were described across patients by MBS status on or after the index date. Continuous variables were reported as mean (standard deviation [SD]) and compared using the t-test. Categorical and binary variables were reported as number (%) and compared using the Chi-square test.

For the primary and secondary continuous outcome analyses, generalized estimating equations were used to evaluate mean systolic and diastolic blood pressure and number of AHMs over time based on time-updated MBS status. For the secondary time-to-event analyses, multivariable pooled logistic regression models were used to estimate the hazard of complete AHM discontinuation and ATRH based on time-updated MBS status. For all analyses, marginal structural modeling with stabilized inverse probability weighting was used to account for time-dependent confounding, with weight-truncation at the first and ninety-ninth percentiles to address extreme weights while avoiding introducing selection bias by excluding those individuals with extreme weights [24]. This approach facilitates adjusting for time-updated covariates that may also be mediators, without adjusting away the relationship between the time-updated MBS and blood pressure outcomes.

We selected baseline and time-updated covariates a priori based on factors known or suspected to potentially influence likelihood to undergo MBS and blood pressure control in patients with obesity. Baseline covariates were age, sex, race, ethnicity, BMI, eGFR, diabetes mellitus, heart failure, arrhythmia, atherosclerotic cardiovascular disease, obstructive sleep apnea, metabolic dysfunction-associated steatotic liver disease, and antihypertensive class [23, 25, 26]. Time-updated covariates were BMI, eGFR, diabetes mellitus, heart failure, arrhythmia, atherosclerotic cardiovascular disease, obstructive sleep apnea, metabolic dysfunction-associated steatotic liver disease, and antihypertensive class. Analyses that did not include systolic or diastolic blood pressure as an outcome were also adjusted for baseline and time-updated systolic and diastolic blood pressures. Effect modification was evaluated with separate models incorporating interaction terms for sex, baseline age, race, baseline obesity severity (BMI 35 – 39.9 vs. ≥40 kg/m^2^), and baseline diabetes status. All analyses were performed using Stata version 18.0 (StataCorp, College Station, TX).

## Results

### Cohort Characteristics

The overall cohort included 187 667 patients with a diagnosis of hypertension and a BMI ≥ 35 kg/m^2^: 183 702 individuals who were managed medically and 3 965 individuals who received MBS (Table 1). Across the entire cohort, the median duration of follow-up was 5.1 (IQR 2.2–9.1) years. In the surgical cohort, the median time to MBS from index date was 4.2 (IQR 1.8–7.4) years with a median duration of follow-up post-MBS of 2.6 (1.0–5.4) years. At baseline, patients who received MBS were more likely to be female (22 vs 10%, p < 0.001). Due to the large sample size, several smaller differences between the cohorts were statistically significant. For example, patients who underwent MBS were slightly younger (54 vs. 55 years, p < 0.001), had slightly lower blood pressures (139 vs. 140 mm Hg systolic and 82 vs. 83 mm Hg diastolic, p < 0.001) and were prescribed slightly fewer AHMs (1.5 vs. 1.6, p < 0.001) at baseline than medical patients. There were small yet statistically significant differences in rates of various obesity-associated comorbidities, which can be seen in Table 1.

**Table 1:** Baseline Characteristics by Bariatric Surgery Status.

Characteristic	Overall N = 187 667	Bariatric Surgery Ever N = 3 965	No Bariatric Surgery N = 183 702	P-Value
Age, years (SD)	55 (11)	54 (11)	55 (12)	<0.001
Female, n (%)	18 669 (10%)	870 (22%)	17 799 (10%)	<0.001
Race/Ethnicity, n (%)				
Black Non-Hispanic	32 679 (17%)	723 (18%)	31,956 (17%)	0.001
White Non-Hispanic	125 897 (67%)	2 686 (68%)	123 211 (67%)	
Hispanic	10 501 (6%)	235 (6%)	10 266 (6%)	
Other or Unknown Race	18 590 (10%)	321 (8%)	18 269 (10%)	
BMI, kg/m^2^ (SD)	39.3 (4.3)	39.1 (4.4)	39.3 (4.3)	<0.001
Systolic blood pressure, mm Hg (SD)	140 (14)	139 (14)	140 (14)	<0.001
Diastolic blood pressure, mm Hg (SD)	83 (10)	82 (10)	83 (10)	<0.001
Estimated glomerular filtration rate, mL/min/1.73m^2^*	87 (19)	89 (19)	87 (19)	<0.001
Chronic kidney disease, n (%)^†^	11 407 (6%)	198 (5%)	11 209 (6%)	<0.001
Diabetes mellitus, n (%)	64 116 (34%)	1 132 (29%)	62 984 (34%)	<0.001
Cardiovascular disease, n (%)	12 166 (6%)	320 (8%)	11 846 (6%)	<0.001
Heart failure, n (%)	5 013 (3%)	86 (2%)	4 927 (3%)	0.047
Arrhythmia, n (%)	16 763 (9%)	394 (10%)	16 369 (9%)	0.025
Peripheral artery disease, n (%)	6 516 (3%)	170 (4%)	6 346 (3%)	0.005
Obstructive sleep apnea, n (%)	17 360 (9%)	232 (6%)	17 128 (9%)	<0.001
Metabolic dysfunction-associated steatotic liver disease, n (%)	9 648 (5%)	263 (7%)	9 385 (5%)	<0.001
Current or prior smoker, n (%)	48 714 (26%)	1 025 (26%)	47 689 (26%)	0.877
Antihypertensive medications, n (SD)	1.6 (0.9)	1.5 (0.8)	1.6 (0.9)	<0.001
Type of Bariatric Surgery, n (%)				
Gastric Bypass		499 (13%)		
Duodenal Switch		593 (15%)		
Sleeve Gastrectomy		215 (5%)		
Gastric Band		27 (1%)		
Unknown Type		2 631 (66%)		

All values are presented as mean (SD) or n (%).

*Estimated using the CKD-EPI 2021 race-free equation.

^†^Determined by eGFR <60 mL/min/1.73m^2^ or presence of proteinuria.

### Blood Pressure Control

**Fig. 2:**
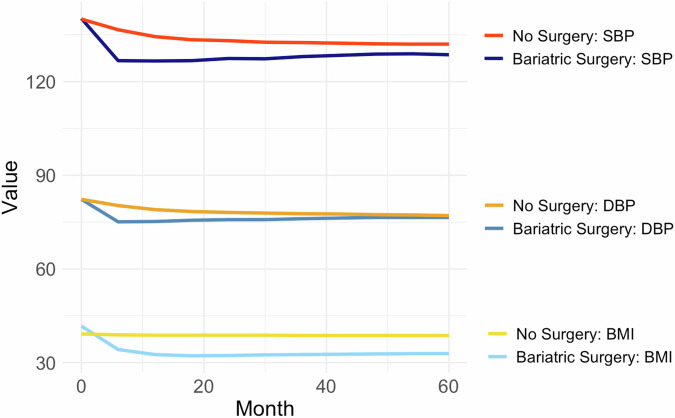
Trends in mean body mass index, systolic blood pressure and diastolic blood pressure over six-month bins in patients managed with bariatric surgery versus medically with a minimum of 5 years of follow-up. Note that “Month 0” represents the mean values from the year prior to bariatric surgery in MBS patients and one year prior to index date in non-surgical patients.

At the index date, both the surgical and medical cohorts demonstrated elevated blood pressures with the same mean systolic blood pressures of 140 mm Hg and mean diastolic blood pressures of 83 mm Hg. Using time-updated analyses that accounted for the varying duration to MBS and to the development of comorbid conditions over time, MBS patients had better blood pressure control throughout the study period. Average systolic blood pressure was 5.4 mm Hg (95% CI -5.9 to -4.9 mm Hg) lower and average diastolic blood pressure was 1.8 mm Hg (95% CI -2.1 to -1.5 mm Hg) lower in those who underwent MBS vs. medical therapy over the duration of follow-up (Table 2). In general, improvements in BMI in the MBS cohort correlated with improvements in blood pressure control (Fig. 2). Patients lost the majority of their excess weight in the first six months after surgery with a corresponding drop in mean blood pressure. Over the following 4.5 years, both BMI and blood pressure measurements remained relatively stable, indicating a durable effect. In non-MBS patients, there was no drop in BMI, though mild improvements in SBP and DBP could be detected. There was no effect modification by sex, age, race, obesity severity, or baseline diabetes status.

**Table 2:** Blood Pressure-Related Outcomes after Bariatric Surgery.

Outcome	β (95% CI)
Systolic blood pressure, mm Hg	−5.4 (−5.9, −4.9)
Diastolic blood pressure, mm Hg	−1.8 (−2.1, −1.5)
Antihypertensive medication classes, number*	−0.5 (−0.86, −0.10)
	**Hazard ratio (95% CI)**
Off antihypertensive medication*	1.32 (1.23, 1.42)
Resistant hypertension*	0.86 (0.78−0.95)

All analyses were adjusted for baseline age, sex, race, and ethnicity as well as baseline and time-updated BMI, eGFR, diabetes mellitus, heart failure, arrhythmia, atherosclerotic cardiovascular disease, obstructive sleep apnea, metabolic dysfunction-associated steatotic liver disease, and antihypertensive classes.

*Also adjusted for baseline and time-updated systolic and diastolic blood pressure.

### Antihypertensive Medication Trajectories

MBS was associated with lower AHM requirements over time when adjusted for time-updated blood pressure control. Patients who underwent MBS were on an average of 0.48 (95% CI -0.86 to -0.10) fewer AHMs during follow-up compared to those who did not undergo MBS. There were 57 354 patients who discontinued AHMs and 64 274 patients who developed ATRH during follow-up. Patients who received MBS had a 32% greater likelihood of discontinuing AHMs entirely (7.0 vs. 5.5 events per 100 person-years; adjusted hazard ratio [HR] 1.32, 95% CI 1.23–1.42) and 14% lower likelihood of developing ATRH (7.3 vs. 9.7 events per 100 person-years; adjusted HR 0.86, 95% CI 0.78–0.95). There was no effect modification by sex, age, or baseline diabetes status.

## Discussion

MBS represents the single most effective intervention for weight loss, with numerous studies endorsing its ability to produce impressive and durable weight loss for patients with obesity [27–29]. While weight loss is indeed a desirable outcome, it is the metabolic effects of weight loss which can produce dramatic changes in patient quality of life; improvement and even resolution in the end-organ impacts of metabolic syndrome such as diabetes and hypertension can be life-altering. In this study, we found that patients with hypertension who underwent MBS experienced significant improvements in BMI in the initial six months following surgery with corresponding improvements in blood pressure control. These improvements were durable out to five years. Importantly, patients who underwent MBS required fewer AHMs, had an over 30% greater likelihood of discontinuing AMH therapy, and a nearly 15% lower likelihood of developing ATRH. These findings support the role of MBS in preventing, treating, and mitigating the cardiovascular comorbidities of obesity by demonstrating improved control of the number one modifiable risk factor for cardiovascular morbidity, hypertension.

This study substantiates the growing body of research supporting the cardiovascular benefits of MBS in the management of obesity. In general, few studies have emphasized hypertension as a primary outcome when assessing MBS, however many have assessed it in secondary analyses. Three meta-analyses of prior research on MBS and cardiovascular outcomes have shown similar improvements in blood pressure control in surgically managed patients, with over a quarter of patients experiencing remission of hypertension and significant decreases in the number of required AHMs [9–11]. However, the majority of studies on this subject lack follow-up beyond the immediate post-operative period and fail to account for the time-dependent bias inherent to matching patients undergoing a procedure to controls. Only a prospective randomized controlled trial, such as the GATEWAY trial, can account for these biases via randomization, but this study is limited in its population size and generalizability [13–15]. Our study validates the impact of MBS on hypertension outcomes by demonstrating these granular improvements in blood pressure control in a large, real-world population over an extended follow-up period. Our study also accounts for differences in timing of MBS as well as the development of comorbid disease over time, both before and after the development of hypertension and concomitant obesity. We were also able to identify lower rates of new onset ATRH in our study, a relatively novel finding otherwise only demonstrated recently by our team in a nationwide claims-based matched cohort study of MBS patients [12].

These findings have significant implications in the management of cardiovascular health outcomes. As obesity rates continue to rise worldwide, it is necessary to take proactive measures to combat this pandemic and its downstream health impacts; the majority of obesity-related deaths are secondary to cardiovascular disease, much of which is mediated via hypertension [30–32]. That MBS can be effective at both reducing excess weight and improving hypertensive outcomes validates its role in improving overall patient wellness and further supports its cost efficacy. Despite high upfront costs, significant downstream morbidity can be prevented across multiple disease spectrums, with substantial cost savings [33]. Policymakers and insurance providers, especially those able to retain patients on their plans for prolonged periods, should heed this robust evidence when approaching decision-making regarding coverage for this “elective” procedure.

This study’s primary strength lies in its extended follow-up period for patients compared to other similar retrospective studies; using the VA’s database, we were able to longitudinally follow patients for many years before and after exposure to MBS and to account for important time-updated factors that may influence the decision to obtain MBS as well as obesity-related hypertension outcomes. However, this database is not without limitations; the VA population represents a very different cohort than the general population seeking MBS. VA patients undergoing MBS were generally older with a much higher skew toward male patients than the general population, somewhat limiting the generalizability of this study [34]. Further, the continuity of care provided by the VA, both in terms of insurance provision and longitudinal follow-up, separates this patient population from generally younger patients electively seeking MBS who may not have access to consistent follow-up. This study was also unable to separate outcomes by specific bariatric procedure (i.e. sleeve gastrectomy versus RYGB) or by the number of procedures performed. The reason for this is that a substantial portion of patients were missing information on the specific type of bariatric surgery performed; the most frequently used ICD codes and internal VHA procedural codes indicate that MBS was performed, but do not include any granularity on the specific surgery performed. Future studies will require individual chart review or natural language processing to determine this level of detail. We are also unable to account for patients who received MBS outside of the VHA, though we would expect this to skew our results toward the null. We also cannot control for patient motivation to pursue weight loss and health-seeking behaviors, which may be increased in patients willing to undergo MBS. Nevertheless, this study represents one of the largest, most granular, and longest duration retrospective assessments of the impact of MBS on hypertension control over time.

Finally, it is important to note that, given the relative novelty of glucagon-like peptide 1 agonists for weight loss, we were unable to assess their impact in this cohort. In this study, improvements in blood pressure over time seemingly correlated with drops in BMI, perhaps implying that loss of excess weight had greater impact on blood pressure control than any other hormonal effects of MBS. Comparing the effect of MBS vs. glucagon-like peptide 1 agonists vs. combination therapy with both modalities on hypertension outcomes will be an important avenue for future research.

## Conclusion

In this large longitudinal retrospective cohort study of patients followed within the VHA, patients with hypertension who underwent MBS had improved hypertension outcomes. These patients demonstrated improvements in BMI and blood pressure control. They had higher rates of AHM discontinuation and lower rates of ATRH. These findings further validate the role of MBS in the management of cardiovascular morbidity in patients with obesity.

## Summary

### What is known about topic

- Obesity is a risk factor for poor blood pressure control.
- Metabolic and bariatric surgery (MBS) is an effective treatment for obesity and metabolic syndrome.
- Patients who undergo MBS are more likely to experience improved blood pressure control, but studies are limited by short duration follow-up.

### What this study adds

- Patients with hypertension who undergo MBS have significantly better blood pressure control than those who do not over prolonged follow-up.
- MBS is associated with higher likelihood of antihypertensive medication discontinuation, and lower likelihood of new onset apparent-treatment resistant hypertension.

## Supplementary information

Supplemental Material

## Data Availability

The VHA Corporate Data Warehouse contains national electronic health record data including detailed demographic, clinical, laboratory, and pharmacy fill records. The VA Informatics and Computing Infrastructure (VINCI) is a partner with the Corporate Data Warehouse and hosts all data available through CDW. For CDW access please contact VINCI@VA.GOV.

## Code availability

Relevant code used for statistical analysis can be made available upon direct request to the study authors.
