# Supplementary material for: The Effect of Bariatric Surgery on Hypertension Outcomes: A Retrospective Cohort Study
Source: J Hum Hypertens. 2025 Aug 20;39(10):683–9. doi: 10.1038/s41371-025-01063-z (PMC12500470; doi:10.1038/s41371-025-01063-z)
Supplement: Supplementary file 1 — Supplemental Material [file 41371_2025_1063_MOESM1_ESM.docx]

**Supplemental Materials for:** **“**The Effect of Bariatric Surgery on Hypertension Outcomes: A Retrospective Cohort Study”

**Authors:** Jesse E. Passman, MD, MSHP, MPH^1,2^; Amanda Bader, MD^1^; Nadim Mahmud, MD, MPH^3^; Kristoffel R. Dumon, MD^1^; Heather Wachtel, MD, MTR^1^; Feibi Zheng, MD, MBA^4,5^, Jordana B. Cohen, MD, MSCE^2,6,7^

**Affiliations:**

1. Department of Surgery, Perelman School of Medicine, University of Pennsylvania, Philadelphia, PA

2. Leonard Davis Institute, University of Pennsylvania, Philadelphia, PA

3. Department of Gastroenterology, Perlman School of Medicine, University of Pennsylvania, Philadelphia, PA

4. Global Health Economics & Outcomes Research Division, Intuitive, Sunnyvale, CA

5. Michael E. DeBakey Department of Surgery, Baylor College of Medicine, Houston, TX

6. Renal-Electrolyte and Hypertension Division, Department of Medicine, Perelman School of Medicine, University of Pennsylvania, Philadelphia, PA

7. Department of Biostatistics, Epidemiology, and Informatics, Perelman School of Medicine, University of Pennsylvania, Philadelphia, PA

**Running Title:** Bariatric Surgery and Hypertension

**Corresponding Author:**

Jesse Passman, MD, MSHP, MPH

[jesse.passman@pennmedicine.upenn.edu](mailto:jesse.passman@pennmedicine.upenn.edu)

Hospital of the University of Pennsylvania

3400 Spruce Street, 4 Maloney Building

Philadelphia, PA 19104

**Table S1. Codes used to identify metabolic and bariatric surgeries and obesity-related comorbidities**

|  | **Code Type** | **Codes** |
| --- | --- | --- |
| **Obesity-Related Comorbidities** | | |
| Hypertension^1^ | ICD-9 | 401.x, 403.0x, 403.1x, 403.9x |
|  | ICD-10 | I10, I12.0, I12.9 |
| Diabetes mellitus^2^ | ICD-9 | 249.xx, 250.0x |
|  | ICD-10 | E08.xx, E09.xx, E10.xx, E11.xx, E13.xx, O24.xx, O99.8xx, P70.2 |
| Heart failure^3^ | ICD-9 | 398.91, 402.01, 402.11, 402.9, 404.x, 428.x, 518.4 |
|  | ICD-10 | I09.81, I11.0, I13.0, I13.2, I50.x, J81.0 |
| Arrhythmia^3^ | ICD-9 | 427.x |
|  | ICD-10 | I46.2, I46.8, I46.9, I47x, I48.x, I49.x, R00.1 |
| Atherosclerotic cardiovascular disease^3^ | CPT-4 | 33510-33523, 33530, 33533-33536, 33542, 33545, 33548 |
|  | ICD-9 | 410.x, 411.1, 412, 413.x |
|  | ICD-10 | I20.x, I21.x, I22.x, I25.x |
| Obstructive Sleep Apnea^4^ | ICD-9 | 327.23 |
|  | ICD-10 | G47.33 |
| Non-Alcoholic Fatty Liver Disease/Non-alcoholic Steatohepatitis^5^ | ICD-9 | 571.8, 571.9 |
|  | ICD-10 | K75.81, K76.0 |
| **Metabolic and Bariatric Surgeries** | | |
| Roux-en-Y Gastric Bypass^6^ | CPT-4 | 43633, 43644-43847 |
|  | ICD-9 Procedure | 44.3, 44.31, 44.32, 44.38, 44.39 |
|  | ICD-10 Procedure | 0D16079, 0D1607A, 0D160J9, 0D160JA, 0D160K9, 0D160KA, 0D160Z9, 0D160ZA, 0D1607A, 0D160JA, 0D160KA, 0D160ZA, 0D1687A, 0D168JA, 0D168K9, 0D168KA, 0D168ZA, 0D168Z9, 0D16879, 0D1687A, 0D168ZA, 0D168J9, 0D16479, 0D1647A, 0D164J9, 0D164JA, 0D164K9, 0D164KA, 0D164Z9, 0D164ZA |
| Sleeve Gastrectomy^6^ | CPT-4 | 43775, 43842 |
|  | ICD-9 Procedure | 43.82 |
|  | ICD-10 Procedure | 0DB64Z3, 0DQ60ZZ, 0DQ63ZZ, 0DQ67ZZ, 0DB60Z3 |
| Biliopancreatic Diversion/Duodenal Switch^4^ | CPT-4 | 43845 |
|  | ICD-9 Procedure | 43.89, 45.51, 45.91 |
|  | ICD-10 Procedure | 0D190Z9, 0DB60ZZ, 0DB80ZZ |
| Adjustable Gastric band^4, 6^ | CPT-4 | 43843, 43770 |
|  | ICD-9 Procedure | 539.01, 539.09, V53.51 |
|  | ICD-10 Procedure | 0DV64CZ |
| Bariatric Revision^6^ | CPT-4 | 43281, 43282, 43332, 43333, 43334, 43335, 43336, 43337, 43771, 43772, 43773, 43774, 43848, 43850, 43855, 43860, 43865, 43886, 43887, 43888 |
|  | ICD-9 Procedure | 44.96, 44.97, V45.86 |
|  | ICD-10 Procedure | 0DW643Z, 0DW64CZ, Z98.84 (converted from ICD-9 Procedure Codes) |
| Bariatric Surgery (non-specific) | ICD-9 Procedure | V45.86 |
|  | ICD-10 Procedure | Z9884 |
|  | VHA Stop Code | 487 |
| Prior Bariatric Surgery* | ICD-10 Diagnosis | Z98. 84 |

*Used to identify ineligible individuals

**Table S2. Classes and generic names of anti-hypertensive medications^7^**

| **Class** | **Drug** |
| --- | --- |
| **First-line agents** | |
| Thiazide or thiazide-type diuretics | Chlorthalidone |
|  | Hydrochlorothiazide |
|  | Indapamide |
|  | Metolazone |
| Angiotensin-converting enzyme inhibitors | Benazepril |
|  | Captopril |
|  | Enalapril |
|  | Fosinopril |
|  | Lisinopril |
|  | Moexipril |
|  | Perindopril |
|  | Quinapril |
|  | Ramipril |
|  | Trandolapril |
| Angiotensin receptor blockers | Azilsartan |
|  | Candesartan |
|  | Eprosartan |
|  | Irbesartan |
|  | Losartan |
|  | Olmesartan |
|  | Telmisartan |
|  | Valsartan |
| Calcium channel blockers—dihydropyridines | Amlodipine |
|  | Felodipine |
|  | Isradipine |
|  | Nicardipine SR |
|  | Nifedipine LA |
|  | Nisoldipine |
| Calcium channel blockers—nondihydropyridines | Diltiazem ER |
|  | Verapamil IR |
|  | Verapamil SR |
|  | Verapamil-delayed onset ER |
| **Second-line agents** | |
| Diuretics—loop | Bumetanide |
|  | Furosemide |
|  | Torsemide |
| Diuretics—potassium sparing | Amiloride |
|  | Triamterene |
| Diuretics—aldosterone antagonists | Eplerenone |
|  | Spironolactone |
| Beta blockers—cardioselective | Atenolol |
|  | Betaxolol |
|  | Bisoprolol |
|  | Metoprolol tartrate |
|  | Metoprolol succinate |
| Beta blockers—cardioselective and vasodilatory | Nebivolol |
| Beta blockers—noncardioselective | Nadolol |
|  | Propranolol IR |
|  | Propranolol LA |
| Beta blockers—intrinsic sympathomimetic activity | Acebutolol |
|  | Penbutolol |
|  | Pindolol |
| Beta blockers—combined alpha- and beta-receptor | Carvedilol |
|  | Carvedilol phosphate |
|  | Labetalol |
| Direct renin inhibitor | Aliskiren |
| Alpha-1 blockers | Doxazosin |
|  | Prazosin |
|  | Terazosin |
| Central alpha2-agonist and other centrally acting drugs | Clonidine oral |
|  | Clonidine patch |
|  | Methyldopa |
|  | Guanfacine |
| Direct vasodilators | Hydralazine |
|  | Minoxidil |

**Supplemental References**

1. Xu Y, Derington CG, Addo DK, He T, Jacobs JA, Mohanty AF *et al.* Trends in Initial Antihypertensive Medication Prescribing Among >2.8 Million Veterans Newly Diagnosed With Hypertension, 2000 to 2019. *J Am Heart Assoc* 2024; **13**(20)**:** e036557.

2. Schroeder E, Donahoo W, Goodrich G, Raebel M. Validation of an Algorithm for Identifying Type 1 Diabetes in Adults Based on Electronic Health Record Data. *Pharmacoepidemiol Drug Saf* 2018; **27**(10)**:** 1053-1059.

3. Cohen JB, Cohen DL, Herman DS, Leppert JT, Byrd JB, Bhalla V. Testing for Primary Aldosteronism and Mineralocorticoid Receptor Antagonist Use Among U.S. Veterans : A Retrospective Cohort Study. *Annals of internal medicine* 2021; **174**(3)**:** 289-297.

4. Elsaid M, Li Y, Bridges J, Brock G, Minacapelli C, Rustgi V. Association of Bariatric Surgery With Cardiovascular Outcomes in Adults With Severe Obesity and Nonalcoholic Fatty Liver Disease. *JAMA Network Open* 2022; **5**(10)**:** e2235003.

5. Hayward K, Johnson A, Horsfall L, Moser C, Valery P, Powell E. Detecting non- alcoholic fatty liver disease and risk factors in health databases: accuracy and limitations of the ICD-10- AM. *BMJ Open Gastro* 2021; **8**(e000572).

6. Arterburn D, Wellman R, Emiliano A, Smith S, Odegaard A, Murali S *et al.* Comparative Effectiveness and Safety of Bariatric Procedures for Weight Loss: A PCORnet Cohort Study. *Annals of Internal Medicine* 2018; **169**(11).

7. Whelton P, Carey R, Aronow W, Casey D, Collins K, Himmelfarb C *et al.* 2017 ACC/AHA/AAPA/ABC/ACPM/AGS/APhA/ASH/ASPC/NMA/PCNA Guideline for the Prevention, Detection, Evaluation, and Management of High Blood Pressure in Adults. *J Am Coll Cardiol* 2018; **71**(19)**:** e127-e248.
